# Immunomodulation and Intestinal Morpho-Functional Aspects of a Novel Gram-Negative Bacterium *Rouxiella badensis* subsp. *acadiensis*

**DOI:** 10.3389/fmicb.2021.569119

**Published:** 2021-06-22

**Authors:** Nour Yahfoufi, Nawal Alsadi, Jean Francois Mallet, Garima Kulshreshtha, Maxwell Hincke, Nafissa Ismail, Chantal Matar

**Affiliations:** ^1^Department of Cellular and Molecular Medicine, Faculty of Medicine, University of Ottawa, Ottawa, ON, Canada; ^2^Department of Innovation in Medical education, Faculty of Medicine, University of Ottawa, Ottawa, ON, Canada; ^3^School of Psychology, Faculty of Social Sciences, University of Ottawa, Ottawa, ON, Canada; ^4^School of Nutrition, Faculty of Health Sciences, University of Ottawa, Ottawa, ON, Canada

**Keywords:** probiotic, mucosal immunity, immuno-modulation, micoRNAs (miRNAs), gastro-intestinal tolerance

## Abstract

A novel bacterium (*Rouxiella badensis* subsp. *acadiensis*) isolated from the microbiota of wild blueberry fruit was investigated for its immunomodulation capabilities and intestinal morpho-functional aspects. The whole-genome shotgun sequencing of this bacterium led to its new taxonomy and showed absence of pathogenicity genes. Although the bacterium was used for blueberry-fermentation and enhancing its anti-inflammatory effects on neurodegeneration, diabetes, and cancer, no study has assessed the effect of the bacterium on health. In this study, we used several *in vitro* and *in vivo* assays to evaluate the interaction of *R. badensis* subsp. *acadiensis* with the intestinal mucosa and its impact on the localized immune response. The strain antibiotic susceptibility has been investigated as well as its tolerance to gastric and intestinal environment and ability to attach to human intestinal epithelial cells (Caco-2 and HT-29). In addition, Balb/c mice were used to explore the immune-modulatory characteristics of the live bacterium at the intestinal level and its impact on the morpho-functional aspects of the intestinal mucosa. *In vitro* assays revealed the ability of *R. badensis* subsp. *acadiensis* to survive the gastric and intestinal simulated conditions and to satisfactorily adhere to the human intestinal epithelial cells. The bacterium was shown to be sensitive to an array of antibiotics. Immuno-modulation studies with mice orally administered with *R. badensis* subsp. *acadiensis* showed a higher number of IgA positive cells in the small intestine, a higher concentration of the anti-inflammatory cytokine IL-10 in the intestinal mucosa, as well as an increase in the number of goblet cells. The anti-inflammatory cytokine miR146a was found to be increased in the ileum and brain. Furthermore, it increases the number of goblet cells which contribute to intestinal barrier integrity. Taken together, our findings reflect the ability of the tested bacterium to modulates the intestinal homeostasis and immune response. Detailed safety unpublished studies and genome data support our finding. The strain *Rouxiella badensis* subsp. *acadiensis* has been filed in a provisional patent; a U.S. Provisional Application No. 62/916,921 entitled “Probiotics Composition and Methods.” Future studies are still needed to validate the potential utilization of this strain as functional food and its potential probiotic effect.

## Introduction

The bacterium *Rouxiella badensis* subsp. *acadiensis* (*R. badensis* subsp. *acadiensis*) explored in this study was previously called *Serratia vaccinii* (Martin and Matar, 2005). The strain was isolated from the surface of lowbush blueberries (Vaccinium angustifolium Aiton) by inoculation of a Tryptic Soy Broth (TSB) (Difco Laboratories, Detroit, MI, United States) with whole blueberry fruit as described in Martin and Matar (2005). In this study, inocula were prepared from a Research Cell Bank of the bacterium. *R. badensis* subsp. *acadiensis* is a Gram negative, catalase positive, facultatively anaerobic coccobacillus. It has a fermentative ability of D-glucose, D-fructose, D-mannose, arbutin, esculin, salicin, saccharose, and D-raffinose and under specific conditions and can ferment mannitol, lactose, and trehalose (Martin, 2003; Martin and Matar, 2005). Functionally, the protective properties of a blueberry-fermented preparation utilizing this bacterium has shown a wide range of anti-inflammatory effects on neurodegeneration, diabetes, and cancer (Vuong et al., 2016, 2007; Nachar et al., 2017; Sánchez-villavicencio et al., 2017). As per published study the bacterium previously referred to as *Serratia vaccinii* has been deposited under Accession Number 160103, at the International Depositary Authority of Canada. They referred to it as Serratia based on its 16S rRNA gene sequence associated with biochemical profile and physical properties (Martin and Matar, 2005). As the 16S rRNA gene sequence analysis was shown to be insufficiently robust to distinguish closely related species and new advanced techniques have emerged, a whole-genome shotgun sequencing and assembly of bacterial strain were performed to determine the phylogenetic position of the isolate. These analyses led to the description of a novel *Rouxiella badensis* subspecies for which we propose the name *R. badensis* subsp. *acadiensis*, following the region where it was firstly isolated. The type strain is Canan SV-53. The novel bacterium has been deposited at the International Depositary Authority of Canada under Accession Number 160103, (Antioxydant Producing Bacterium and USES Therefore US 8,617,870). A taxonomy description paper of the strain is now under revision.

Unpublished data related to genome propose a potential probiotic activity of the bacterium since full genome analysis showed that there are no genes found coding for pathogenicity. A US Provisional Application No. 62/916,921 entitled “Probiotics Composition and Methods” has been filed related to the bacterium. Since there are more papers in the process dealing with the bacterium toxicology and safety uses, we have evaluated in this study some *in vitro* and *in vivo* characteristics of the Gram negative *R badensis subsp. acadiensis* and its impact on immunomodulation and intestinal homeostasis and mucosal immunity.

Our aim is to study the bacterium ability to interact with the intestinal mucosa and modulate the immune response in order to determine whether future analyses are worth it to investigate potential probiotic activity. In the future, we will be proposing probiotic activity once the safety studies will be published especially that *Rouxiella* belongs to the family of Yerciniacae. While recent findings reveal beneficial immunological effects of certain dead bacteria (Vinderola and Reinheimer, 2003; Mottet and Michetti, 2005), most studies emphasize the importance of colonizing the intestinal epithelium to sustain bacterial beneficial effects (Gopal et al., 2001; Kos et al., 2003; Ouwehand and Salminen, 2003; Kumura et al., 2004). Thus, the assessment of bacterial tolerance to human gastric or intestinal juice is of great importance to see if the alive bacterium can reach the intestine (Sanders and Huis In’t Veld, 1999; Shah, 2001). Furthermore, beneficial bacteria can adhere to intestinal mucosal surfaces, thus adherence is a key property that shows the ability of bacteria to avoid elimination, to better colonize the intestinal epithelium and to compete with other microorganisms comprising pathogens for nutrients and attachment sites (Kos et al., 2003). In the host, a complex relationship exists between commensal bacteria, their metabolites, the gut mucosal barrier and the immune and non-immune cells along the intestine (Yahfoufi et al., 2018). The mucosal layer is an important physical barrier; it contains goblet cells which secrete a highly glycosylated mucin and create a net-like layer playing a key role in barrier maintenance. Moreover, they can produce anti-microbial proteins, chemokines, and cytokines with major role in innate immunity. Oral administration of probiotics strengthens the intestinal barrier integrity: they can influence the gut barrier by numerous mechanisms including modulation of mucus production, enhancement of tight junctions, induction of IgA and modulation of the immune response (Hemaiswarya et al., 2013; Scully et al., 2013). Moreover bacteria can modulate proinflammatory and anti-inflammatory cytokines (Ren et al., 2013; Boonma et al., 2014; Elian et al., 2015). For example, *Lacticaseibacillus rhamnosus*, *Bifidobacterium longum*, *Lactobacilus acidophilus*, *Lactobacillus ruteri*, *Lacticaseibacillus paracasei*, are able to modulate the T cells response by up-regulating T reg cells and IL-10 levels in intestinal contents of mice by indirectly involving short-chain fatty acids (SCFAs; Atarashi et al., 2013) which are produced by anaerobic bacterial fermentation of prebiotics (Yahfoufi et al., 2018).

Secretory IgA is of fundamental importance in host mucosal protection against mucosal pathogens (Perdigon et al., 2001; Perdigón et al., 2002). Numerous studies have reported the role of IgA+ cells in maintaining homeostasis of the intestine (Vinderola G. et al., 2005; Galdeano et al., 2007; Mallet et al., 2014). Hence the importance to investigate the effect of *R. badensis subsp. Acadiensis* on the number of IgA positive cells in Peyer’s patches and in the lamina propria as well as on the production of secretory IgA.

Furthermore, small non-coding RNAs regulate gene expression post-transcriptionally (Zhou et al., 2017). miRNAs have been shown to regulate various biological processes involved in inflammation, immune homeostasis and intestinal integrity (Gaudet et al., 2017). Certain miRNAs could initiate an anti-inflammatory response such as miR145 and miR146a while other miRNAs play a role in proinflammatory response and some have mixed immunomodulatory effects (Bermudez-Humaran and Langella, 2017). Bacteria like probiotics were found able to modulate miRNAs (O’Connell et al., 2012).

Finally, it is important to address concerns that have been raised in the use of some bacterial strains that carry antibiotic resistance genes themselves, as they may pass the antibiotic resistance genes to pathogenic bacteria through horizontal gene transfer. Therefore, antibiotic resistance is often studied in bacterial characterization which can present a challenge to their safe usage. Screening bacterial strains for antibiotic resistance is important to ensure their safety and reduce their potential in contributing to the spread of antibiotic resistance genes (Imperial and Ibana, 2016).

Although the most commonly used bacteria for their potential health benefits are Gram-positive (G+) bacteria such as lactic-acid producing bacteria and other that belong to the genus Bifidobacterium, some Gram-negative (G-) bacteria like *Escherichia coli Nissle* 1917(EcN) can exert health efficacy (Saad et al., 2013). When comparing G- and G+ probiotics, G- probiotics show higher activity in reducing the levels of inflammatory mediators during enteric infections (Cross et al., 2004). Referring to existing studies, G- induce higher IL10-responses than G+ probiotics resulting in differential induction of antibody responses. G- EcN has more potent immuno-stimulatory effects than G+ *Lactobacillus rhamnosus* GG in term of inducing mucosal and systemic B cells and results in higher production of IgA at the small intestinal level (Hessle et al., 2000; Kandasamy et al., 2016). The microbe-associated molecular patterns differ between G- and G+ probiotics and can explain the difference in induction of IL-10. In addition, higher antibody response induced by G- when compared to G+ probiotics can be due to different modulation of cytokine milieu (Karlsson et al., 2002; Smits et al., 2004). It is well known that variations in immunomodulation characteristics are strain-dependent even within G+ probiotics (Christensen et al., 2002).

Therefore, we investigated the resistance of this bacterium to commonly used antibiotics, its tolerance to simulated gastric and pancreatic juices, and ability to adhere to intestinal epithelial cells. *In vivo*, the bacterium was administered to Balb/c mice: IL-6, IL-10, and secretory IgA concentrations in mice intestinal fluid were measured, in addition to the abundance of IL-10 positive cells in the ileum. Moreover, we investigated the morpho-functional changes in the small intestine, and the expression of selected anti-inflammatory miRNAs (miR145 and miR146a).

## Materials and Methods

### Bacterial Cultures and Media

Stock cultures of the bacterium were maintained at −80°C in Tryptic Soy Broth (Difco Laboratories, Detroit, MI, United States) supplemented with 30% (v/v) glycerol. The bacterium was grown in Tryptic Soy Agar (TSA) (Difco Laboratories, Detroit, MI, United States) or Tryptic Soy Broth (TSB) (Difco Laboratories, Detroit, MI, United States) at 30°C for 20 h.

### Determination of Antibiotic Resistance

Overnight bacterial culture inocula were diluted 1:50 in fresh TSB and grown until the optical density at 600 nm reached 0.2 (∼10^8^ CFUs/ml). Thereafter, the standardized inoculum (100 μl, OD_600_ = 0.2–10^8^ CFUs/ml) was spread uniformly on TSA. The antibiotic sensi-disks (BD BBL, Sensi-Disc, Becton, Dickinson and Company, United States) used were the following:

(ampicillin:10 μg, Tetracyclin: 10 μg, streptomycin: 10 μg, chloramphenicol: 30 μg, erythromycin: 15 μg or neomycin: 5 μg). Blank disks used as negative control were obtained as well from (BD BBL, Sensi-Disc, Becton, Dickinson and Company, United States). While ciprofloxacin 5 μg, trimethoprim-sulfamethoxazole 25 μg were obtained from Oxoid, ON, Canada. Antibiotic disks or blank disks were placed at the center of the TSA plate (one disk per plate). The plates were incubated at 37°C until even growth of the bacterium was observed. The zones of growth inhibition were measured using a Vernier caliper to categorize the strains as susceptible or resistant to the antibiotic. Experiments were performed three times in duplicates.

### Determination of Tolerance to Simulated Gastric Juices and Simulated Pancreatic Juices

#### Preparation of Simulated Gastric Juice

Simulated gastric juices were prepared by suspending pepsin (Sigma, Saint Louis, MO, United States) to a final concentration of 3 g/l in sterile NaCl (Sigma, Saint Louis, MO, United States) 0.5% weight to volume and adjusting the pH to 2.0, 3.0 or 4.0 with concentrated HCl or sterile 0.1 mol/l NaOH (Huang and Adams, 2004).

#### Preparation of Simulated Pancreatic Juice

Simulated pancreatic juice was prepared by suspending porcine pancreatin (Sigma, Saint Louis, MO, United States) in sterile saline to a final concentration of 1 g/l, with 0.45% bile salts (Sigma, Saint Louis, MO, United States), and adjusting the pH to 7 or 8 with concentrated HCl or sterile 0.1 mol/l NaOH (Huang and Adams, 2004).

#### Determination of Tolerance to Simulated Gastric and Pancreatic Juices

A volume of bacterial inoculum corresponding to 10^8^ to 10^9^ CFU/ml from an overnight culture was subjected to low-speed centrifugation 5000 × *g* for 5 min and the pellet was washed two times in 1X PBS (137 mM NaCl 2.7 mM KCl 10 mM Na_2_HPO_4_, 1.8mM mM KH_2_PO_4_) pH7. The starting count was determined by standard plate count prior to assay transit tolerance. When screening for gastric or intestinal transit tolerance, 1 ml of simulated gastric juice (pH 2, 3, or 4) or 1 ml of simulated pancreatic juice (pH 7 or 8) and 300 μl of NaCl (0.5% W/V) were added to washed bacterial suspension in 2 ml screw cap sterile microcentrifuge tubes. The preparations were vortexed at low speed for 10 s and incubated at 37°C. When testing for gastric transit tolerance, aliquots of 100 μl were collected after 60, 90, and 180 min for the determination of total viable counts; while aliquots of 100 μl were collected after 60, 90, 180, and 240 min when screening for intestinal transit tolerance. To better imitate the passage through the gastro-intestinal tract, bacterial inoculum exposed to simulated gastric juice (pH 2 or 4) for 180 min was centrifuged and re-exposed to simulated pancreatic juice (pH 7 or 8) for 240 min and bacterial count was done after collecting aliquots of 100 μl. Each 100 μl was serially diluted, spread on TSA plates, and incubated at 30°C for 72 h, followed by determination of CFU count. Results are expressed as Log_10_ of the CFU/ml.

### Adhesion to Epithelial Intestinal Cell Lines

#### Cell Cultures

Caco-2 cells (ATCC-HTB-37) were grown in minimum essential medium alpha (MEMα; Gibco Canada, Burlington, ON, Canada) containing 10% v/v inactivated fetal bovine serum (FBS; Thermo Fisher Scientific), 1% non-essential amino acids (Gibco) and a mixture of antibiotics at a final concentration of 50 IU/ml penicillin and 50 μg/ml streptomycin. Cells were cultured at 37°C in a humidified atmosphere of 5% v/v CO_2_. For adherence and invasion assays, confluent Caco-2 cells were harvested by trypsinization in 0.01% EDTA. Caco-2 cells were adjusted to 10^5^ cells/ml by counting in a hemocytometer, seeded in a 24-well tissue culture plate and incubated at 37°C in a 5% CO_2_ humidified atmosphere until a confluent monolayer formed. Prior to adhesion assay, the cell monolayers were washed twice with 1X PBS (pH 7.4-Sigma, Saint Louis, MO, United States) to remove the antibiotics.

HT-29 (ATCC-HTB-38) cell line was maintained in McCoy’s medium (Gibco, United States) supplemented with 10% (v/v) heat inactivated bovine fetal serum and a mixture of antibiotics at a final concentration of 50 IU/ml penicillin, 50 μg/ml streptomycin. Cells were grown at 37°C, 5% CO_2_. Culture media were changed every two days and the cell line was trypsinized with 0.25% trypsin-EDTA solution (Sigma, Saint Louis, MO, United States). For experiments, 10^5^ cells/ml were seeded in 24-well plates and incubated to confluence. Caco-2 cells were incubated till 15 days old while HT-29 were incubated as per Arboreya et al. to confluence during 13 ± 1 days.

#### Adhesion Assay

Adhesion assay was explored as per Arboleya et al. (2011); bacterial cultures were harvested by centrifugation, washed twice with 1X PBS buffer (Sigma, Saint Louis, MO, United States) and resuspended in MEMα or McCoy’s medium supplemented with 10% heat inactivated bovine fetal serum without antibiotics. Prior to adhesion assay, the cell monolayers of Caco-2 or HT-29 were washed three times with 1X PBS (pH 7.4-Sigma, Saint Louis, MO, United States) to remove the antibiotics before adding bacterial suspensions. Bacterial cells were inoculated into wells containing confluent monolayers of Caco-2 or HT-29 cells at a targeted multiplicity of infection (MOI) of 100:1. The actual numbers of bacteria in the inoculum added to the monolayers were confirmed retrospectively by serial dilution and plate counting. Bacteria-infected cells were incubated for 4 h at 37°C and 5% CO_2_ to allow for bacterial adherence. Supernatants were discarded and wells were gently washed three times with 1X PBS buffer to remove the non-attached bacteria. After gentle scraping of cells, the bacterial counts were carried out by plating on TSA to determine the number of adhering bacteria. Serial dilutions were plated and incubated for 48 h at 30°C. The adherence (expressed as a percentage) was calculated by using the ratio of the number of bacterial cells that remained attached to the total number of bacterial cells added initially to each well.

### Animals and Bacterial Supplementation

Six to eight-week-old Balb/c female mice weighing 20–25 g were obtained from Charles River (Montreal, Canada). A total of 20 mice were divided into experimental and control groups consisting of 10 mice per group for all experiments except for the qPCR of brain and ileum (21 mice were used further details are provided below). Pairs of mice were housed together in plastic cages kept in a controlled atmosphere (temperature 22 ± 2°C) with a 12 h light/dark cycle. Mice received 10^8^ CFU of *R. badensis* subsp. *acadiensis*/mouse by gavage for 7 consecutive days in 1% sucrose, 1X PBS pH 7.4 (sigma). Control mice received the same volume of 1% sucrose in 1X PBS instead. All mice received simultaneously a conventional balanced diet *ad libitum* and water. Test and control animals were sacrificed after 7 days of probiotic administration. All experimental procedures were approved by the Animal Care Committee of the University of Ottawa (protocol ME-2403-R3) in accordance with guidelines established by the Canadian Council of Animal Care.

### Bacterial Translocation Assay

After 7 days, treated and control animals were anesthetized and sacrificed by cervical dislocation; livers were immediately harvested under sterile conditions and homogenized using the Omni TH homogenizer with a 7-mm generator probe (Omni International, Kennesaw, GA, United States) in 5 ml sterile 1X PBS pH 7.4 (Sigma, St. Louis, MO, United States). One milliliter of the liver homogenate was spread onto the surface of MacConkey agar for enterobacteria (Difco Laboratories, Detroit, MI, United States). The plates were then aerobically incubated at 37°C for 48 h. Translocation was considered to have occurred when colonies were observed on the agar plates because the liver is an organ normally devoid of bacteria (Gregory et al., 1996).

### Histological Studies of the Gut

The small intestines of sacrificed mice were removed, washed and the ileum parts were collected and fixed overnight in 4% PFA solution (paraformaldehyde). Afterward, the tissues were dehydrated in increasing concentrations of alcohol, cleared in xylene, and embedded in paraffin wax using conventional methods (Alturkistani et al., 2016). Then histological slices of 4μm were prepared from paraffin blocks using a rotation microtome (Leica RM2255 Automated Microtome). Processing, paraffin embedding, and histological slices were performed by The University of Ottawa histology core facility.

#### B Population (IgA+ and IgG+ Cells) Identification by Immunofluorescence

The number of IgA and IgG producing (IgA+ and IgG+) cells was determined on histological slices prepared from the ileum using the direct immunofluorescence method. The immunofluorescence test was performed using (α-chain) anti-mouse IgA FITC conjugate (Sigma–Aldrich, St. Louis, MO, United States) or (δ-chain) anti-mouse IgG FITC conjugate (Sigma–Aldrich, St. Louis, MO, United States). Histological sections were deparaffinized and then rehydrated in a graded ethanol series. Sections were incubated at 37°C in two different solutions of xylene (Sigma–Aldrich, St. Louis, MO, United States) successively for 15 min in each solution followed by incubation in graded ethanol series 95, 70, and 40% for 5 min each at 4°C. Deparaffinized histological samples were incubated with the appropriate antibody dilution (1:100 for IgA or 1:50 for IgG) in 1X PBS solution for 30 min at 37°C in the dark. Samples were then washed two times with PBS solution and examined using a fluorescent light microscope. Dry slides were mounted using Fluoromount (Sigma-Aldrich, St. Louis, MO, United States). The results were expressed as the number of IgA+ or IgG+ cells (positive = fluorescent cell) per 10-fields (magnification 100×). Data represent the mean of three readings for each animal.

#### Determination of IL-10+ Cells in Mice Small Intestine

At the end of the 7 days period, the small intestine was removed and processed for histological preparation as described above. The indirect immunofluorescence method was used to label IL-10 positive cells. After deparaffinization of the histological slices, they were rehydrated in a graded series of ethanol -as per described above- and then incubated for 30 min in a 1% blocking solution of bovine serum albumin (Jackson Immuno Research, West Grove, PA, United States) at room temperature. Histological slices mounted on slides were then incubated for 60 min at 37°C with rabbit anti-mouse IL-10 polyclonal antibodies (Peprotech, Inc., Rocky Hill, NJ, United States) at a dilution 1:50. 1X PBS solution was used for two consecutive washes, then sections were treated for 45 min at 37°C with a 1:100 dilution of a goat anti-rabbit antibody conjugated with FITC (Jackson Immuno Research, West Grove, PA, United States). Finally, sections were washed twice with 1X PBS and examined using a fluorescent light microscope. Dry slides were mounted using Fluoromount (Sigma-Aldrich, St. Louis, MO, United States). The results were expressed as the number of IL10 + cells (positive = fluorescent cell) per 10-fields (magnification 100X).

#### Determination of Small Intestine Morpho-Functional Changes

Different intestinal segments were collected from treated and control animals.

Intestinal segments were fixed in 4% PFA, dehydrated, cleared and embedded in paraffin. Serial sections were cut at 4 μm, deparaffinized in xylene, rehydrated, and stained with hematoxylin and eosin. For staining neutral glycoconjugates or acid glycoconjugate in the intestinal sections we used periodic acid Schiff (PAS) or Alcian blue (AB), pH 2.5. This staining reveals neutral PAS reactive and acid AB-reactive glycoconjugates (Smirnov et al., 2005; Martín et al., 2016). All processing, paraffin embedding and histological staining with hematoxylin, eosin, PAS or AB were performed by The University of Ottawa histology core facility.

The morphology of different intestinal segments was revealed by staining with hematoxylin and eosin for examination by light microscopy. Positive goblet cells for neutral glycoconjugates or acid glycoconjugates were counted. The results were expressed as the number of goblet cells per 10-fields (magnification 100×). Data represent the mean of three readings for each animal.

### Determination of IL-6, IL-10, and Secretory IgA in Mice Intestinal Fluid

The small intestine of treated and control animals was flushed with 5 ml of 1X PBS pH7.4 (Sigma, St. Louis, MO, United States) and this fluid was centrifuged at 10,000 *g* at 4°C for 10 min to separate particulate material. The supernatant was collected and kept frozen at −80°C until use. IL-6 and IL-10 were determined in supernatant using the corresponding mouse ELISA Set (BD OptEIA, BD Biosciences PharMingen, San Diego, CA, United States) with catalog numbers respectively: 555240 and 555252. For IL-6, capture antibody was diluted at 1:250 in coating buffer (BD OptEIA, BD Biosciences PharMingen, San Diego, CA, United States) while the detection antibody was diluted at 1:259 in assay diluent (BD OptEIA, BD Biosciences PharMingen, San Diego, CA, United States). For Il-10, dilutions were respectively 1:250 dilution in coating buffer for capture antibody while 1:250 dilution in assay diluent for the detection antibody. For secretory IgA, the level of IgA was analyzed by DAS-ELISA using affinity-purified goat anti-mouse IgA antibodies (α-chain specific) was added at 1.25 mg/well and horseradish-peroxidase conjugated anti-IgA specific antibodies at 1.25 mg/well (Sigma Chemical Co., St Louis, MO, United States). Absorbance was read at 450 nm within 30 minutes of stopping reaction with wave length correction 570 nm.

### Real-Time Quantitative Reverse Transcription PCR

Ileum pieces and brains from 11 control: CTR and 10 *R. badensis* subsp. *acadiensis* treated mice: *R. badensis* subsp. *acadiensis* were snap frozen after collection and kept at −80°C until RNA extraction. Samples were extracted using miRNeasy mini kit (Qiagen, Toronto, ON, Canada). Sample purity were verified with a NanoDrop 2000 (Thermo Scientific, Waltham, MA, United States). Samples underwent a reverse transcription reaction to produce cDNA using individual probes. The cDNA was synthesized by Moloney Murine Leukemia Virus (MMLV) Reverse Transcriptase (Invitrogen, Canada inc.). The expression of target anti-inflammatory miR-145 and miR-146a was measured by RT-qPCR using Taqman primers for hsa-miR-146a (Thermofisher Taqman miRNA assay ID 000468) and hsa-miR-145 (Thermofisher Taqman assay ID 002278) and a FastStart Taq Polymerase (Roche, Mississauga, ON, Canada) in a CFX96 real-time PCR detection system (Bio-RAD, Laboratories, Hercules, CA, United States). Gene expression was normalized to gene reference U6 small non-coding RNA (Thermofisher assay ID 001973).

## Statistical Analysis

Results are presented as means ± SEM. Results represent three independent experiments. GraphPad Prism 5.0 software (GraphPad Software Inc., San Diego, CA, United States) was employed to carry out statistical analysis. The statistical significance was determined by applying an unpaired two-tailed Student’s *t*-test when comparing two groups, and one-way analysis of variance (ANOVA) when comparing more than two groups. A difference was considered significant for *p*-values <0.05.

## Results

### Antibiotic Susceptibility by Agar Disk Diffusion Method

*Rouxiella badensis* subsp. *acadiensis* demonstrated susceptibility to different classes of antibiotics like β-lactams, fluoroquinolones, aminoglycosides, chloramphenicols, tetracyclines, and trimethoprim-sulfamethoxazole. *R. badensis* subsp. *acadiensis* is sensitive to ampicillin 10μg (a β-lactam) with a diameter of inhibition zone (di) greater than 17 mm di = 38.67 ± 0.72), the strain is sensitive to ciprofloxacin 5 μg (fluoroquinolones class) with a di = 39.16 ± 1.57 mm > 21 mm. Streptomycin 10 μg (an aminoglycoside) revealed an inhibition zone with a di = 18.08 ± 0.73 > 15 mm reflecting sensitivity of the strain. The bacterium is as well sensitive strain to: chloramphenicols 30 μg with a di = 23.5 ± 1.38 > 15 mm, tetracyclines 10 μg with di = 27.6 ± 1.1 > 15 mm and trimethoprim-sulfamethoxazole 25 μg with a di = 24.5 ± 2.1 > 16 mm while it possessed intermediate sensitivity to erythromycin15 μg (a macrolide) with a di = 20.1 ± 0.6 mm (Table 1).

**TABLE 1:** Antibiotic susceptibility of *R. badensis* subsp. *acadiensis* to different antibiotics using the agar disk diffusion method.

Antibiotic disk	Class of the antibiotic	Diameter (d) of inhibition zone in mm*	Zone diameter (mm) indicating			Conclusion
			
			S	I	R	
Ampicillin 10μg	β-lactams	38.67 ± 0.72	≥17	14–16	≤13	S
Ciprofloxacin 5 μg	Fluoroquinolones	39.16 ± 1.57	≥21	16–20	≤15	S
Streptomycin 10 μg	Aminoglycosides	18.08 ± 0.73	≥15	12–14	≤11	S
Chloramphenicol 30 μg	Chloramphenicols	23.5 ± 1.38	≥15	13–17	≤12	S
Trimethoprim-sulfamethoxazole 25 μg	Folate pathway antagonists	24.5 ± 2.1	≥16	11–15	≤10	S
Tetracycline 10 μg	Tetracyclines	27.6 ± 1.1	≥15 (30 μg)	12–14	≤11	S
Erythromycin 15 μg	Macrolides	20.1 ± 0.6	≥23	14–22	≤13	I

*Comments: S (sensitive), I (intermediate sensitivity), R (resistant).*

### Determination of Tolerance to Simulated Gastric Juices and Simulated Pancreatic Juices

**FIGURE 1:**
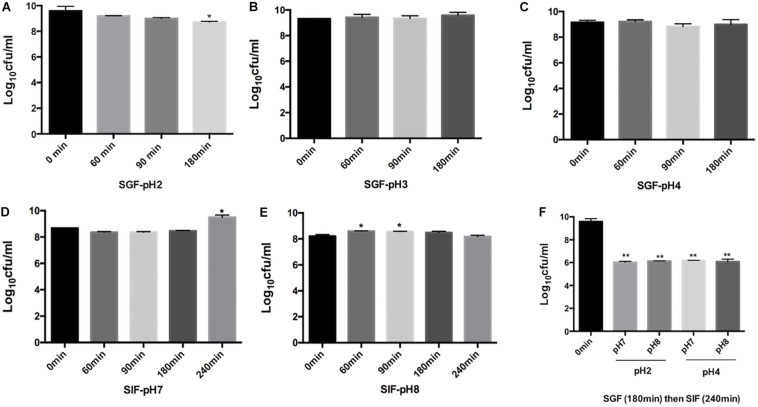
Effect of simulated gastric juices SGF [pH 2 **(A)** pH 3.0 **(B)**, pH 4.0 **(C)**] and simulated intestinal juice SIF [pH7 **(D)**, pH 8 **(E)**] on the viability of *R. badensis* subsp. *acadiensis* that was exposed to SGF for 60, 90,and 180 min and to SIF for 60, 90,180, and 240 min. In panel **(F)** *R. badensis* subsp. *acadiensis* was first exposed to SGF for 180 min then to SIF for 240 min. Data represents mean log10CFU/ml ± SEM (*n* = 3). Significant difference exists if *p < 0.05, **p < 0.01.

*Rouxiella badensis* subsp. *acadiensis* showed high stability when incubated in simulated gastric juice. The total duration of incubation tested was 180 min at 37°C which is equivalent to time the bacteria are usually resident at the stomach level. The studies showed that the bacterium displayed high survival and high tolerance to the gastric juice at pH 3 and 4 (Figures 1B,C). After incubation for 180 min at pH 2 we observed a significant killing effect *p* < 0.05 (Figure 1A). However, the bacterium was still able to survive at high rate.

*Rouxiella badensis* subsp. *acadiensis* tolerated the simulated pancreatic juices as well; survival percentages were tested after exposure for 240 min. The bacterium showed highest survival and viability when incubated at pH 7 for 240 min. At pH 8, bacterial growth was observed after 60- and 90-min incubation at 37°C but after 3 and 4 h incubation there were no difference with *t* = 0 min. The bacterium maintained high survival rate at simulated pancreatic juice (Figures 1D,E). After passage of the strain through simulated gastric (pH 2 or 4) for 180 min and subsequent intestinal conditions (pH 7 and 8) for 240 min, we observed higher killing ability of the same tested pHs previously tested (Figure 1F).

### Adhesion to Human Epithelial Intestinal Cell Lines

Adhesion to cells of the intestinal epithelium is another property that probiotics must possess for successful colonization of the human gastrointestinal tract.

**FIGURE 2:**
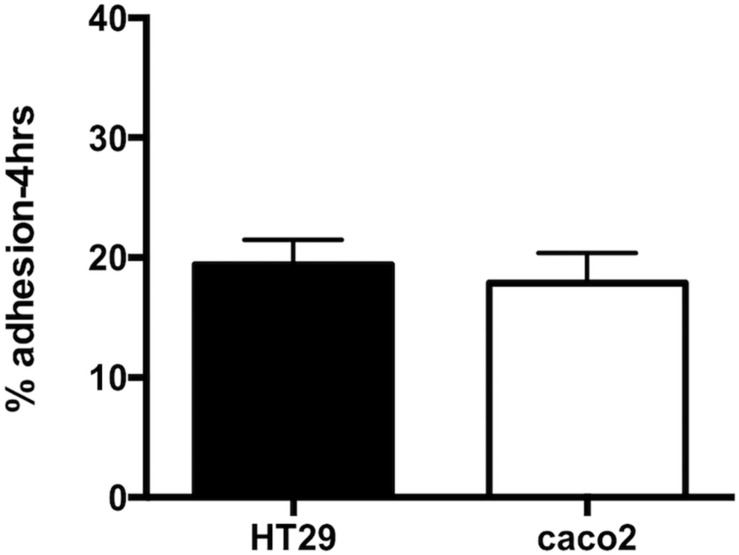
Data represents percentage of adhesion of *R. badensis* subsp. *acadiensis* to HT29 and Caco2 cells. Results shown are means of three independent experiments ± SEM.

The ability of *R. badensis* subsp. *acadiensis* to adhere to intestinal epithelial cells was studied *in vitro*. Human epithelial colorectal adenocarcinoma Caco-2 and HT-29 cells were used. An adhesion rate of 17.8% ± 2.516 on Caco-2 cells, and 19.92% ± 2.078 on HT29 cells was found (Figure 2; Turpin et al., 2012; Argyri et al., 2013).

### Bacterial Translocation

Oral administration of *R. badensis* subsp. *acadiensis* did not cause translocation of Enterobacteriacae *to* the liver in mice treated with 10^8^CFU/mouse. No bacterial growth on the MacConckey agar plates was observed for extracts of liver samples collected from Balb/c mice fed the bacterium.

### Histological Studies of the Gut

#### Small Intestine Overall Morphology

The histological study of the small intestine of mice that received *R. badensis* subsp. *acadiensis* for 7 consecutive days showed no edema or mucosal atrophy, compared to control mice. The overall morphology of the small intestine did not show any significant changes when compared to control mice (not shown).

#### B Cells Population (IgA+ and IgG+ Cells)

The mucosal immunomodulating capacity of *R. badensis* subsp. *acadiensis* was assessed in this study by examining its effects on the IgA+ and IgG+ B cell populations and selected cytokines (pro-inflammatory cytokine IL-6 and anti-inflammatory cytokine IL-10) in both the gut mucosa and the intestinal contents.

**FIGURE 3:**
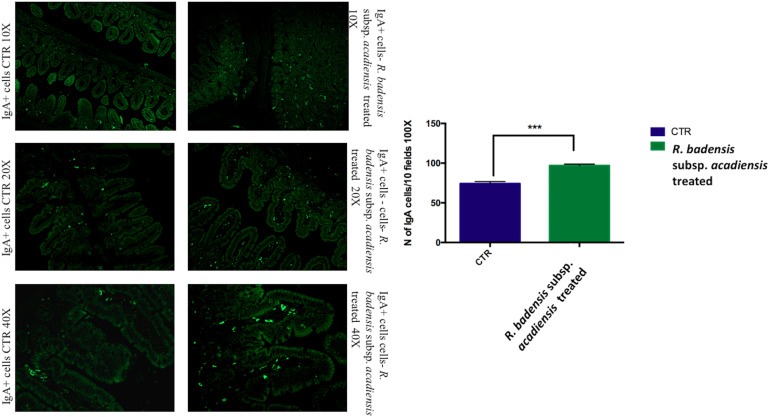
Mean ± SEM of the number of IgA positive cells populations in 10 fields of objective 100X in the ileum of mice fed 1% sucrose (CTR) or *R. badensis* subsp. *acadiensis* fed 10^8^CFU/mouse/day for 7 days. Significant difference exists if *p < 0.05, representative tissue photos are taken with 10×, 20×, and 40 × magnifications.

**FIGURE 4:**
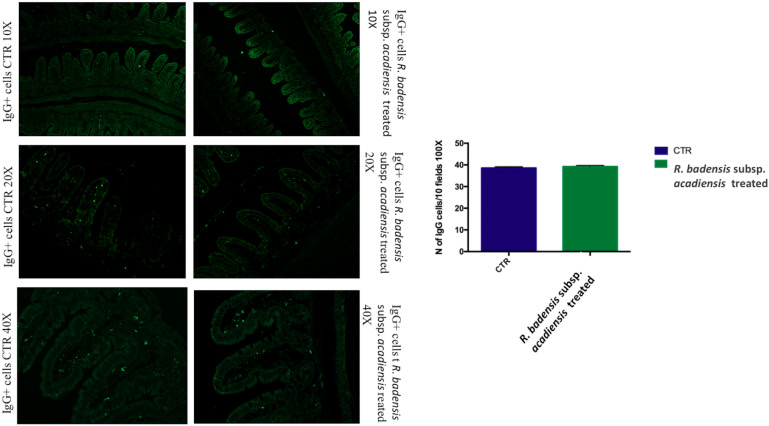
Mean ± SEM of the number of IgG positive cells populations in 10 fields of objective 100X in the ileum of mice fed 1% sucrose (CTR) or *R. badensis* subsp. *acadiensis* fed 10^8^CFU/mouse/day for 7 days. Significant difference exists if *p < 0.05, representative tissue photos are taken with 10×, 20×, and 40 × magnifications.

The number of IgA and IgG producing cells (IgA+ and IgG+) in the lamina propria of the small intestine of mice that received *R. badensis* subsp. *acadiensis* showed a significant increase in the number of IgA+ cells in the lamina propria (Figure 3) (96.89 ± 1.933) in comparison to the control group (74.23 ± 2.588). The number of IgG+ cells was unchanged (Figure 4).

#### Increase of IL10+ Cells in the Ileum

**FIGURE 5:**
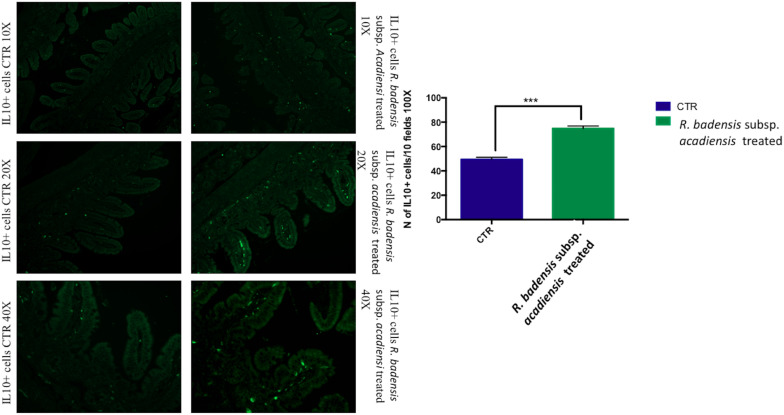
Mean ± SEM of the number of IL10 positive cells populations in 10 fields of objective 100X in the ileum of mice fed 1% sucrose (CTR) or *R. badensis* subsp. *acadiensis* fed 10^8^CFU/mouse/day for 7 days. Significant difference exists if *p < 0.05- *p* is < 0.001 representative tissue photos of IL10 positive cells in the lamina propria are taken with 10×, 20×, and 40 × magnifications.

IL10+ cells were shown to be significantly increased in the intestine of mice receiving the probiotic bacterium (74.83 ± 2.065) versus (49.43 ± 1.805) in the control group (Figure 5).

#### Positive Goblet Cells Determination

**FIGURE 6:**
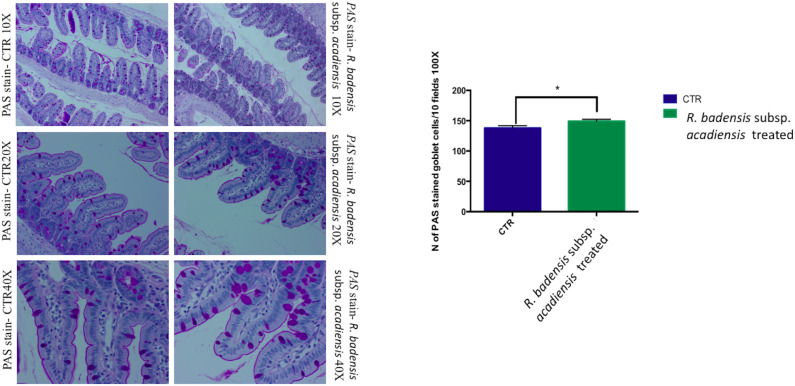
Mean ± SEM of the number of goblet cells stained with PAS (Periodic Acid Schiff) in 10 fields of objective 100× in the small intestine of mice fed 1% sucrose (CTR) or *R. badensis* subsp. *acadiensis* fed 10^8^CFU/mouse/day for 7 days. Significant difference exists if *p < 0.05-*p* = 0.035 Representative tissue photos are taken with objective 10×, 20×, and 40× magnifications of the PAS-stained tissue.

**FIGURE 7:**
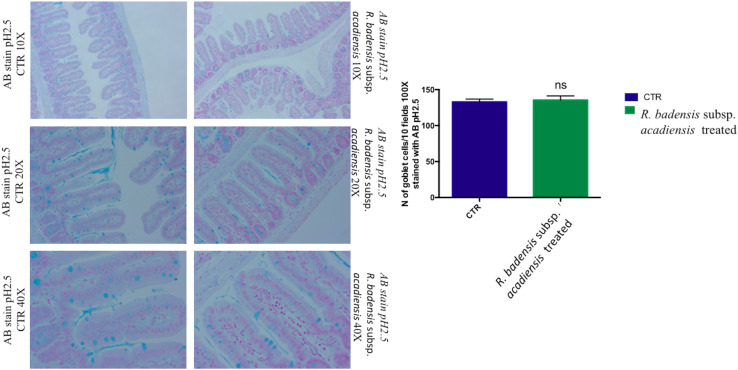
Mean ± SEM of the number of goblet cells stained with AB (Alcian Blue) at pH 2.5 in 10 fields of objective 100× in the small intestine of mice fed 1% sucrose (CTR) or *R. badensis* subsp. *acadiensis* fed 10^8^CFU/mouse/day for 7 days. Significant difference exists if *p < 0.05-Representative tissue photos are taken with objective 10×, 20×, and 40× magnifications of the PAS-stained tissue. AB stains in blue acid glycoconjugates.

Considering the multiple functions of the glycoconjugates that make up intestinal mucins, positive goblet cells for neutral glycoconjugates or acid glycoconjugate were counted after staining intestinal sections with periodic acid Schiff (PAS) and AB (Smirnov et al., 2005; Martín et al., 2016). Tissue histology for small intestine of *R. badensis* subsp. *acadiensis* fed groups displayed a significant increase in the number of PAS stained goblet cells (149.1 ± 3.167) in comparison to the control group (137.9 ± 3.811) with *p* = 0.0355(Figure 6). However, AB staining at pH2.5 did not show any increase in the number of goblet cells positive for acid glycoconjugates (Figure 7).

### IL-10, IL-6, and Secretory IgA Determination

**FIGURE 8:**
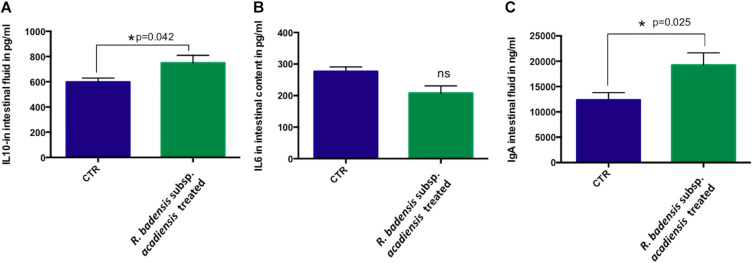
Data represents mean ± SEM of concentration of different **(A)** anti-inflammatory cytokines IL-10, **(B)** pro-inflammatory cytokine-IL-6 as well a **(C)** IgA in the intestinal fluid of (CTR) control mice fed 1% sucrose or *R. badensis* subsp. *acadiensis* treated group 10^8^CFU/mouse/day of *R. badensis* subsp. *acadiensis* for 7 days. Number of animals per group is *n* = 10. Difference is considered significant between groups if *p < 0.05 ns = non-significant difference when *p* > 0.05.

A significant increase in IL-10 was observed in the intestinal fluid of animals that received *R. badensis* subsp. *acadiensis* for 7-days when compared with control mice (*p* = 0.042) (Figure 8A). There was no change in the levels of IL-6 (Figure 8B). Secretory IgA were shown to be increased significantly in the intestinal fluid of mice fed the bacterium (19,232 ng/ml ± 2,431) compared to the control group (12,368 ± 1,429) with *p* = 0.0255 (Figure 8C).

### miR145 and miR146a Expression at the Intestinal and Brain Levels

**FIGURE 9:**
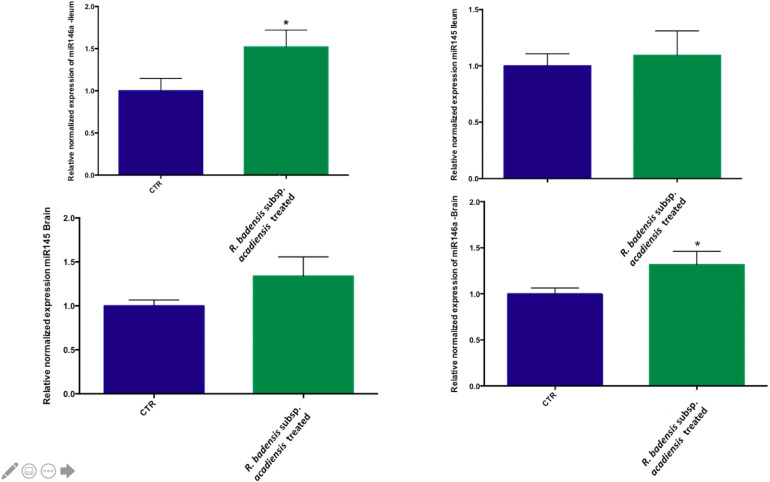
Mean ± SEM of relative expression of miR145 and miR146a in the brain and ileum of (CTR) control mice fed 1% sucrose or *R. badensis* subsp. *acadiensis* treated group 10^8^CFU/mouse/day of Serratia vaccinia for 7 days. Significant difference between mice exists if *p < 0.05.

Although no statistically significant changes were observed in the expression of miR145 in ileum or brain, we did observe a trend toward increased expression in the brain. However, a significant increase in miR146a expression was observed in ileum and brain revealing the anti-inflammatory potential of *R. badensis* subsp. *acadiensis* (Figure 9).

## Discussion

Probiotics are defined as “live microorganisms that, when administered in adequate amounts, confer a health benefit on the host” (Hill et al., 2014). They are identified mainly from fermented foods. Although fermented dairy products remain a major source of probiotic bacteria such as lactic acid bacteria and bifidobacteria; some can also be found in fermented vegetable and fruits products (Battcock et al., 1998; Schrezenmeir and De Vrese, 2001). For example, the native microbiota of vegetables involve a wide variety of lactic acid bacteria species implicated in fermentation (Swain et al., 2014). In this study, we characterize the *in vitro* and *in vivo* ability of *R. badensis* subsp. *acadiensis* isolated from the surface of lowbush blueberries (Vaccinium angustifolium Aiton) and used in blueberry-fermented preparation (Antioxydant Producing Bacterium and USES Therefore US 8,617,870) to survive the gastro-intestinal conditions, interact with the lamina propria, modulate the intestinal barrier integrity, affect mucus production and modify the immune response. Since bacteria can act as potential reservoirs for antimicrobial resistance genes, potential bacterial candidates for human and animal feeds should be screened for antimicrobial resistance. The resistant micro-organisms present in food products may result in infections that are difficult to treat. The disk diffusion method showed that *R. badensis* subsp. *acadiensis* does not carry antimicrobial resistance to the various examined antibiotics. Therefore, the bacterium can be considered as safe concerning resistance to antibiotics.

Resistance to human gastro-intestinal transit represents an important *in vitro* selection criterion to determine the ability of the strain to survive in the intestine and interact with the intestinal mucosa (Goldin et al., 1992). The tested strain must survive passage through the upper gastrointestinal (GI) tract and arrive alive at its site of action in order to better function in the gut environment. In our study, the *in vitro* methodology was used to simulate *in vivo* gastric and small intestinal transit in the presence of bile salts. The investigated strain demonstrated high tolerance to both simulated gastric (pH 3 and 4) containing pepsin and pancreatic juices containing pancreatin and bile salts when tested separately. Nevertheless, the bacterium was more sensitive to simulated gastric juice (pH2) and the exposure to gastric conditions and subsequent intestinal fluid makes it a lot more vulnerable with a survival rate after exposure to both conditions more than 50%. These findings revealed the ability of a good portion of the delivered bacterium to survive in the intestine.

Adhesion to the intestinal epithelial cells permits to assess the ability of the investigated strain to colonize the intestinal mucosa (Fuller et al., 1992; Dunne et al., 2001; Teitelbaum and Walker, 2002). The correlation between intestinal mucosal adhesion and transient colonization is clear between *in vitro* and *in vivo* adhesion in experimental animals as found with several strains of microorganisms (Zárate et al., 2002). A good correlation was reported between the *in vitro* adhesion to Caco-2 cells and the *in vivo* persistence of *Lactobacillus* strains and *Bifidobacterium* strains (Crociani et al., 1995; Jacobsen et al., 1999). Although the ability of a microorganism to adhere to the intestinal mucosa may provide a mean by which the probiotic can modulate the immune system (Helgeland and Brandtzaeg, 2000) and are grounds for competitive exclusion between microorganisms for nutrients and binding sites (Alderberth et al., 2000; Fons et al., 2000), it is now accepted that modulation of immune system and antimicrobial effects could occur independently of the adhesion capabilities of the bacterium. Under the conditions used in our experiment, we have provided evidence that *R. badensis* subsp. *acadiensis* is an *in vitro* adherent strain when tested on Caco-2 and HT-29 cells.

Moreover, the introduction of an intestinal immunomodulating bacteria in the diet must be accompanied by the absence of an adverse inflammatory response (Perdigon et al., 2001). Bacterial translocation or the passage of viable indigenous bacteria from the gut to the mesenteric lymph nodes, liver and other organs can be promoted by disruption of the ecological gastrointestinal equilibrium or disrupted permeability of the intestinal barrier (Berg, 1992; Vinderola C. G. et al., 2005). In the present study, we performed the translocation assay in the liver of mice after the oral administration of *R. badensis* subsp. *acadiensis*; no translocation was observed. Non-pathogenic commensal microorganisms interact closely with the gut epithelial surface and play a key role in the physiological, anatomical and immunological development of the host. Epithelial cells are the first line of defense; they interact simultaneously with bacteria and bacterial products and neighboring immune cells on their basolateral side. They can distinguish between pathogenic and non-pathogenic microorganisms which is an essential characteristic for the tolerance of the normal microbial microbiota (Galdeano et al., 2005; Vinderola C. G. et al., 2005).

Multiple defense mechanisms are implicated in the surveillance of intestinal mucosal homeostasis. Secretory IgA antibodies and mucus secretions are examples of these mechanisms (Fagarasan and Honjo, 2003). In the gut, IgA+ cells and secretory IgA play a key role as the first line of defense in the host. IgA antibodies cooperate with the innate non-specific defense mechanisms, by exerting immune exclusion (Vinderola et al., 2006) and preventing the invasion and colonization of pathogenic microorganisms through competition for receptors and/or metabolic substrates. We observed that the oral administration of *R. badensis* subsp. *acadiensis* increased the number of IgA + cells in the small intestine lamina propria of mice as well as the secretory IgA content in the intestinal lumen. On the other hand, IgG+ cells and the pro-inflammatory cytokine IL-6 did not increase ruling out persistent immunopathology in the intestinal mucosa (Vinderola et al., 2006).

Mucosal barriers established by intestinal epithelial cells maintain gut homeostasis; to better decipher the beneficial effect of *R. badensis* subsp. *acadiensis* strain on mucus producing cells two different specific stains (AB pH2.5 and PAS) were used. PAS staining representative of tissue histology of small intestine of treated and control groups revealed the increase of goblet cell mucus producing cells in mice gavaged with *R. badensis* subsp. *acadiensis*, pointing out a positive effect of the strain on intestinal mucosa. Goblet cells are responsible for the production and preservation of a protective mucus blanket by synthesizing mucins resulting in a dynamic protective barrier. They constitute, along with epithelial cells, macrophages, and dendritic cells, the main cellular components of the innate defense system (Nakata et al., 2017). Mucin glycoproteins synthesized and secreted by goblet cells are major constituents of the mucus layer which functions as a dynamic defensive barrier (Gaskins, 1998; Mack et al., 1999).

Additionally, mucosal immune response homeostasis can be regulated by the immunoregulatory cytokine interleukin 10 (IL-10) that limits mucosal immune responses and minimizes immunopathology (Glocker et al., 2009; Christopher et al., 2013). In fact, mice with a conditional deletion of IL-10 in the CD4+ T cell subset develop spontaneous inflammation of the intestine (Roers et al., 2004; Rubtsov et al., 2008). During acute mucosal infections, the lack of IL-10 can be protective since it is accompanied with an enhanced inflammatory response and an increased production of pro-inflammatory cytokines like IL-12, TNF, and other cytokines (Arai et al., 1995; Gazzinelli et al., 1996; Dai et al., 1997; Ke and Costa, 2006; Dann et al., 2014). However, the absence of IL-10 could result in inflammation (Gazzinelli et al., 1996; Hunter et al., 1997; Reis e Sousa et al., 1999; Suzuki et al., 2000). IL-10 plays a key role in the context of acute bacterial infection of the intestine in preventing excessive inflammation by limiting innate immunity. Similarly, mice deficient for IL-10 or IL-10 receptor developed inflammation of the large intestine (Kühn et al., 1993; Spencer et al., 1998; Yen et al., 2006). Therefore, IL-10 is crucial for maintaining a balance in adaptive immune responses in the mucosae (Denning et al., 2007; Murai et al., 2009; Ueda et al., 2010; Medina-Contreras et al., 2011; Rivollier et al., 2012). Recent studies showed that IL-10 signals drive macrophages to express tolerogenic functions that prevent colitis (Shouval et al., 2014; Zigmond et al., 2014). Our data showed that *R. badensis* subsp. *acadiensis* increased the number of IL-10+ cells in the lamina propria. Secreted IL-10 was also increased in the intestinal fluid. These findings suggest that *R. badensis* subsp. *acadiensis* may actively contribute to mucosal tolerance and may have anti-inflammatory properties (Lee and Mazmanian, 2010).

The role of microRNAs in driving or controlling inflammation is now subject to intense research. More specifically, anti-inflammatory miR-146a is a key regulator of the inflammatory processes involved in Tregs and IL-10 control. The expression of anti-inflammatory molecules like IL-10 is promoted by miR146a overexpression leading to a reduction of the inflammation (Dharap et al., 2009; Liu et al., 2016). On the other hand, recent studies found that the loss of miR-145 stimulates proinflammatory signals in the innate immune system (Akao et al., 2006). In the brain, it plays a role in antioxidant defense (Witwer et al., 2010). Our data supports the ability of *R. badensis* subsp. *acadiensis* to increase the levels of miR146a at the intestine and the brain; this finding highlights its anti-inflammatory activity and its link to IL-10 expression in the ileum.

Our findings reflect the ability of the bacterium to interact with the intestinal mucosa and to modulate the immune response and the gut barrier function. Future papers assessing the bacterium safety will be published in order to assess a potential probiotic activity of *R. badensis* subsp. *acadiensis.*

## Conclusion

We demonstrated that *R. badensis* subsp. *acadiensis* is a non-invasive bacterium. It can survive gastrointestinal tract conditions and has resistance to bile salts and digestive enzymes (pancreatin and pepsin). Under the conditions tested, the bacterium exhibits adhesion properties to Caco-2 and HT-29. The immunoregulatory effects of the novel strain was shown by an increase in IL-10, IgA and miR-146. We conclude that *R. badensis* subsp. *acadiensis* participates in specific local immune protection and that its oral administration enhances gut-associated non-specific immunity without an inflammatory outcome. This strain is a good candidate for further investigation to elucidate its potential health benefits.

## Data Availability Statement

The raw data supporting the conclusions of this article will be made available by the authors, without undue reservation.

## Ethics Statement

The animal study was reviewed and approved by Animal Care Committee (ACC)- university of Ottawa.

## Author Contributions

NY: experimental design, experiments optimization and running, writing the manuscript, data collection and data analysis. CM: main supervisor, revision of experiments and guidance, data review, and manuscript review. MH and GK: collaboration on antibiotic experiments and correction of the manuscript. NA and JM: helps with samples collection during the Euthanasia day. All authors contributed to the article and approved the submitted version.

## Conflict of Interest

The authors declare that the research was conducted in the absence of any commercial or financial relationships that could be construed as a potential conflict of interest.
